# Relational autonomy in the care of the vulnerable: health care professionals’ reasoning in Moral Case Deliberation (MCD)

**DOI:** 10.1007/s11019-017-9818-6

**Published:** 2017-12-14

**Authors:** Kaja Heidenreich, Anders Bremer, Lars Johan Materstvedt, Ulf Tidefelt, Mia Svantesson

**Affiliations:** 1 Faculty of Health and Medicine, University Health Care Research Center, Örebro University, Box 1613, 701 16 Örebro, Sweden; 2 Department of Health and Caring Sciences, Faculty of Health and Life Sciences, Linnaeus University, Växjö, Sweden; 3 PreHospen – Centre for Prehospital Research, University of Borås, Borås, Sweden; 4 Department of Philosophy and Religious Studies, Faculty of Humanities, Norwegian University of Science and Technology (NTNU), Trondheim, Norway; 5 Glasgow End of Life Studies Group, School of Interdisciplinary Studies, University of Glasgow, Dumfries Campus, Scotland, UK

**Keywords:** Clinical ethics, Ethics consultation, Moral case deliberation, Health care professionals, Qualitative research

## Abstract

In Moral Case Deliberation (MCD), healthcare professionals discuss ethically difficult patient situations in their daily practice. There is a lack of knowledge regarding the content of MCD and there is a need to shed light on this ethical reflection in the midst of clinical practice. Thus, the aim of the study was to describe the content of healthcare professionals’ moral reasoning during MCD. The design was qualitative and descriptive, and data consisted of 22 audio-recorded inter-professional MCDs, analysed with content analysis. The moral reasoning centred on how to strike the balance between personal convictions about what constitutes good care, and the perceived dissonant care preferences held by the patient. The healthcare professionals deliberated about good care in relation to demands considered to be unrealistic, justifications for influencing the patient, the incapacitated patient’s nebulous interests, and coping with the conflict between using coercion to achieve good while protecting human dignity. Furthermore, as a basis for the reasoning, the healthcare professionals reflected on how to establish a responsible relationship with the vulnerable person. This comprised acknowledging the patient as a susceptible human being, protecting dignity and integrity, defining their own moral responsibility, and having patience to give the patient and family time to come to terms with illness and declining health. The profound struggle to respect the patient’s autonomy in clinical practice can be understood through the concept of relational autonomy, to try to secure both patients’ influence and at the same time take responsibility for their needs as vulnerable humans.

## Background

Internationally, there is a consensus that healthcare professionals need clinical ethics support to be able to manage ethical issues in daily practice (Abma et al. 2010; Dauwerse et al. 2011). One kind of clinical ethics support service is Moral Case Deliberation (MCD). In MCD, a facilitator-led group of healthcare workers discuss an ethically difficult patient situation taken from daily practice. The role of the facilitator is to support an open dialogue and stimulate the reflection process of the group (Stolper et al. 2015). The purpose of the MCD is to support healthcare professionals in their management of such cases, and the overarching goal is to improve the quality of patient care (Molewijk et al. 2008a, b).

MCD has mostly been conducted and studied in the Netherlands (Dauwerse et al. 2014; Spronk et al. 2017). So far, research has mainly focused on the implementation of the method itself, and on the education of facilitators (Molewijk et al. 2008a, b; Plantinga et al. 2012; Stolper et al. 2015; Weidema et al. 2013). A limited number of Swedish healthcare institutions practice MCD—commonly called ‘ethics rounds’ or ‘ethic case reflections’ (Bartholdson et al. 2014; Gronlund et al. 2016; Silen et al. 2016; Svantesson et al. 2008a, 2014). Swedish studies have utilized a mix of methods to evaluate MCD (Grönlund 2016; Kälvemark Sporrong 2007; Silen et al. 2015; Svantesson et al. 2008b) and various different theoretical models of the discussion have been described in the literature (Hansson 2002). Still, knowledge of the actual content of the discussions is limited.

Recently, the European Moral Case Deliberation Outcome instrument (Euro-MCD) was developed, which measures the desired and experienced outcomes of MCD (Svantesson et al. 2014). As a part of the Euro-MCD project, 70 Swedish MCDs were audio recorded and the facilitators interviewed, in order to understand the content and outcomes of MCD.

So far, the ethically difficult situations raised in these 70 MCDs have been identified (Rasoal et al. 2015), and the facilitators’ experiences of their role in MCD have been described (Rasoal et al. 2017). A quantitative assessment of the content of the dialogue revealed that moral reasoning composed a median of 45% of the spoken time, while reflection on the psycho-social work environment constituted 29% (Svantesson et al. 2017).

However, there is, as mentioned, limited knowledge regarding the content of the moral reasoning that takes place during MCD and further knowledge of the moral reasoning may shed light on ethical deliberation in the midst of clinical practice. Furthermore, the moral content may inform the development of valid outcome criteria for MCD, which are lacking (Hem et al. 2015; Metselaar et al. 2017). Thus, the aim of the present study was to describe the content of healthcare professionals’ moral reasoning during moral case deliberations regarding ethically difficult patient situations.

## Methods

### Design

This study was a qualitative, descriptive study utilizing content analysis according to Elo and Kyngäs (2008).

### Setting and participants

**Fig. 1:**
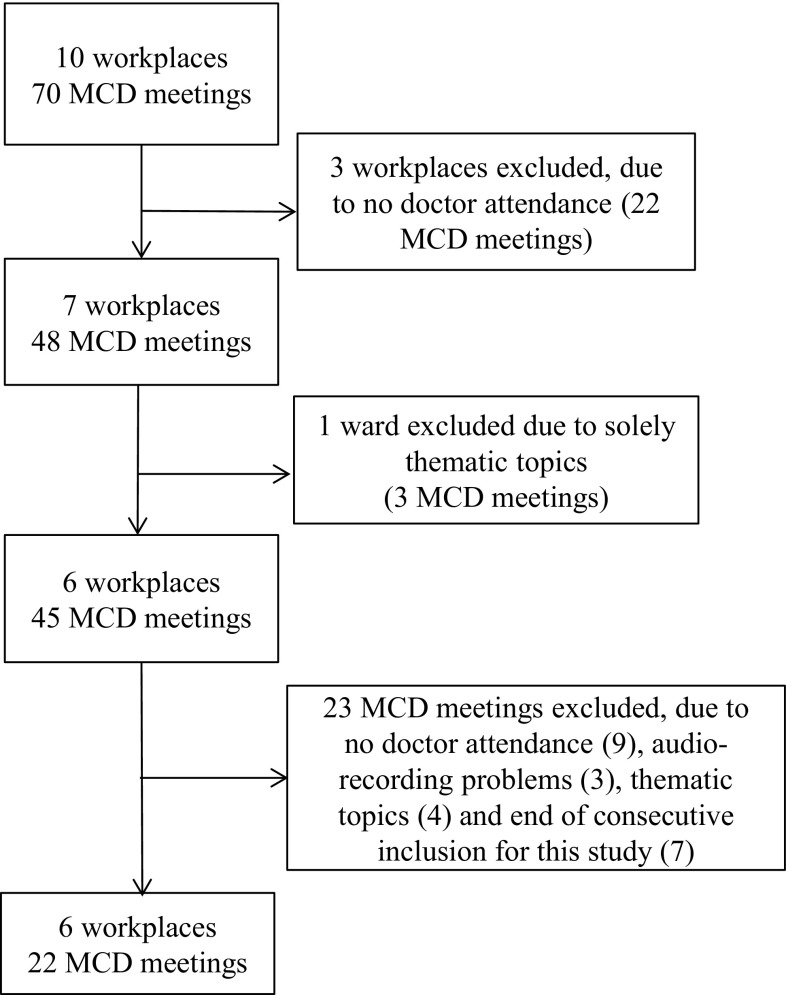
Selection of MCDs

Institutions in mid-Sweden that had access to MCD facilitators were recruited for the larger Euro-MCD project (Svantesson et al. 2014). Inclusion was based on local access to a facilitator, and that the majority of employees had expressed a wish to be supported in ethically difficult situations. Furthermore, the management would have to guarantee that time would be given for participation. The institutions differed in size, and workplaces included general internal medicine, dialysis and geriatric care. For the current study, exclusion criteria were MCDs without doctor participation and thematic session not based on real patient cases, which resulted in 22 audio-recorded MCDs (Fig. 1). The rationale for setting these exclusion criteria was that both the medical and nursing perspectives were secured, and the need to facilitate a deeper analysis compared with the previous study of all 70 MCDs, based on the assumption that patient situations better reflect everyday care (Svantesson et al. 2017).

The MCDs were thus inter-professional and consisted of nurses, nurse assistants, doctors, physiotherapists, and social workers. Nurses comprised the dominant profession. Facilitators were philosophers, chaplains, doctors and supervising nurses (Table 1), whose experiences of their role has been published elsewhere (Rasoal et al. 2017). Facilitators were instructed to assist healthcare staff to reflect systematically on an ethically difficult situation that they had encountered in their daily work (Svantesson et al. 2014). They were also presented with the following definition of an ethically difficult situation: “a situation in which you experience unease or uncertainty about what is right or good to do, or there is disagreement about what should be done” (Svantesson et al. 2014).

**Table 1:** Participating units

Unit	Type of institution	Specialty	Type of facilitator	Number of MCDs	Number of participants Mean (range)
Unit 1	Community hospital	Dialysis care	Philosopher	6	6 (5–8)
Unit 2	Community hospital	Internal medicine	Philosopher	5	10 (8–13)
Unit 3	University hospital	Dialysis care	Two clinical supervisor nurses	3	12 (9–14)
Unit 4	District hospital	Internal medicine	Chaplain and deacon	2	8 (8–9)
Unit 5	Community hospital	Geriatric rehabilitation	Physician	2	8 (8)
Unit 6	Community hospital	Geriatric rehabilitation	Philosopher	4	8 (8–9)
Total				22	9 (5–14)

### Data collection

MCD groups gathered on a monthly basis, on eight occasions in each workplace. Sessions lasted between 60 and 90 min. Facilitators were responsible for the audio recordings, which make up 24 h of recorded discussions. The audio-recordings were transcribed verbatim by an experienced research secretary, yielding data material of 517 pages in total.

### Data analysis

Content analysis was conducted according to (Elo and Kyngas 2008), and facilitated by the software program NVivo 10. All transcripts and audio recordings were read, and respectively listened to, in order to grasp a sense of the whole.

Initially, a deductive approach was applied, in an attempt to describe perspectives from principle-based and relational-oriented ethics (including virtue ethics and ethics of care, as well as ethics of proximity) in the data (Svantesson et al. 2017). This approach was, however, found to be too theoretical and thus failed to allow us to describe the essence of the moral reasoning. Instead, data were coded using an inductive approach, from the perspective of moral reasoning.

In the next step, codes that shared similar meanings according to the aim of the study were merged into generic categories. Codes and categories were continuously validated in view of the data, moving between the parts and the whole for the purpose of refining categories. When writing up the results, re-categorisation continued in the light of the whole. This element of the results is presented under the heading *content*, and is a latent analysis, according to Elo and Kyngas (2008). In trying to grasp how the discussions unfolded on a structural level, we utilized a more manifest approach. Transcripts were re-read with the aim of describing common *structures* of how the discussions unfolded, and data were re-coded according to this aim. The progress of each step in the analysis was scrutinized and discussed between the first and last authors, as well as being co-assessed by the second and third authors, until final agreement was reached both on structure and content in the data.

### Ethical considerations

An advisory statement specifying no objections to the study was issued by the Swedish Regional Ethical Review Board of Uppsala (ref.nr 2012/34). Participants were informed about the research project verbally in meetings as well as in writing. Consent to being recorded was assumed by virtue of participation in the MCD sessions. No names or persons can be identified in the data, and published quotations are only identifiable by the persons who were present.

## Results

**Fig. 2:**
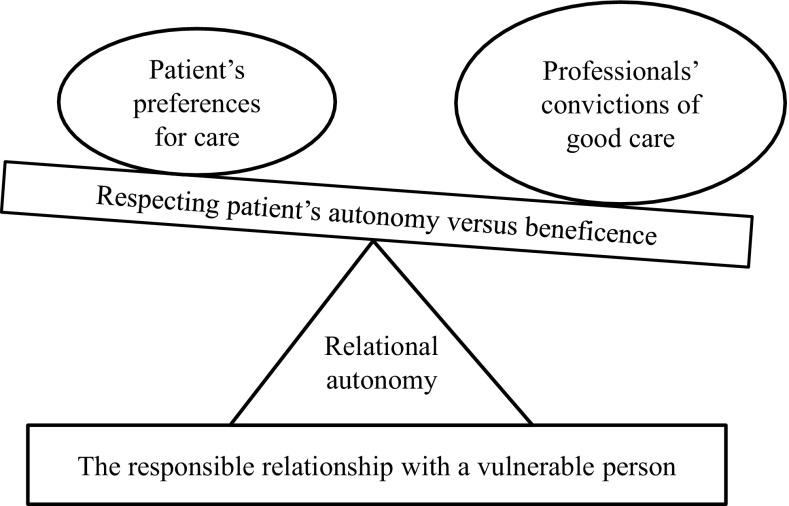
Relational autonomy in the struggle to uphold dignity in illness

The content of healthcare professionals’ moral reasoning during moral case deliberations contained the professionals’ deliberations of how to strike a balance between their convictions about what constitutes good care and the dissonant preferences for care held by the patient, and how to establish a responsible relationship with the vulnerable person. The relationship between the main-categories is illustrated in Fig. 2.

To understand the context of the moral reasoning that occurs within MCDs, the structure of the dialogue is presented first. The structure varied between meetings, workplaces and facilitators; nonetheless, a pattern could be detected. In some MCDs, the main task was to understand exactly what the ethical issue was. In other MCDs, the focus was on discussing possible courses of action or how to cope with the ethically difficult situation.

MCDs began by presenting an inventory of the different situations or problems that the staff wanted to discuss. The facilitator’s role during the initial phase was to help the staff formulate the ethical issues based on the participants’ narratives. During this process the facilitator asked for facts about the patient’s situation. These facts included the patient’s medical history, nursing needs and psychosocial situation, the family’s perspective, and the staff’s previous communication with the patient. Throughout the dialogue, the discussion continuously returned to these facts as well as to assumptions about the patient. The professionals made assumptions about the patient’s and their family members’ behaviour, their emotional needs, and the various possible consequences of the different courses of action. Altogether, facts and assumptions were used for the drafting of the ethical problem, which could be altered after the adding of new facts and assumptions. In all meetings, the healthcare professionals expressed that they had shared experiences and views during the moral reasoning. However, the facilitators seemed to have a key role beyond structuring the dialogue, to provide a critical voice, often adopting the perspective of the absent patients and their families. The facilitators also introduced theoretical concepts that could shed light on the question at hand. They utilized ethical principles, such as “The Four Principles of Biomedical Ethics” as well as ethical concepts having to do with responsibility, vulnerability and power.

The *content* of the moral reasoning was captured in two main categories: “How to strike a balance between convictions of what constitutes good care and the perceived dissonant preferences for care held by the patient”; and “How to establish a responsible relationship with the vulnerable person” (Table 2).

**Table 2:** Results with main and generic categories

Main categories	Generic categories
How to strike a balance between convictions of what constitutes good care and the perceived dissonant preferences for care held by the patient	Framing the notion of good care in relation to demands from patient and family regarded to be unrealistic
	Querying with to what extent it is justifiable to influence the patient’s decision-making in order to achieve good care
	Struggling with standing up for the incapacitated patient’s nebulous interests
	Coping with the conflict between using coercion to achieve good while protecting human dignity
How to establish a responsible relationship with the vulnerable person	Acknowledging the patient as a susceptible human being in a psychosocial context
	Guarding the patient’s dignity and integrity through practical measures in care
	Defining personal moral responsibility towards the patient
	Having patience to give the patient and family time to come to terms with illness and declining health

## How to strike a balance between convictions of what constitutes good care and perceived dissonant preferences for care held by the patient

### Framing the notion of good care in relation to demands regarded as unrealistic from the patient and their family

The professionals expressed strong beliefs about what sort of care was in the patient’s best interest, based on their judgements about the patient’s medical and nursing needs. During the discussions they returned to what they considered to be necessary and adequate care. This was a starting point for the reasoning about how to manage conflicting needs and wishes. Few doubts were expressed about what, in their perspective, ought to be done, nor were any expressed about what it meant to do good for the patient.

I have told the wife and the daughters that we make sure he’s not in pain, make sure he’s not anxious and not having a hard time with his breathing. We make him comfortable. That’s my main priority. (Dialysis care)

The staff’s convictions about what constitutes good and adequate care ran into ethical difficulties, however, when the patient’s or the family’s preferences or wishes diverged from those of the professionals. Participants discussed how to deal with patients who demanded care; a situation that they felt could lead to harm or undesirable consequences for the patient. The demands could relate to discharge, despite the need for more social care or services at home, or demands for inappropriate medication. The potential consequences could include insecurity at home or addiction to narcotic drugs because of inappropriate medication. Both participants and facilitators emphasized that striving to achieve a good outcome for the seriously ill patient must be balanced against the patient’s right to influence their own care.

Some patients were described as demanding support and attention to such an extent that the staff felt uncertain about how to practically frame the care, both for the demanding patient and for the other affected patients on the unit. This could be, for example, a patient who expressed a need for a high degree of presence of the staff due to anxiety or psychiatric comorbidities. The professionals judged which care they should prioritize, and how the needs of one patient had to be balanced against those of other patients in the unit.

In this situation I feel that I really would need to give more time. However, this takes time from the other patients who have needs as well. Then you get bad conscience and unconsciously feel an inner stress. (Geriatric rehabilitation).

### Querying to what extent it is justifiable to influence the patient’s decision-making in order to achieve good care

Participants struggled with situations where the patients refused the particular care that was offered. Professionals and facilitators discussed to what extent it is morally acceptable to influence patients to try to achieve what they judged to be good care, based on their professional assessment of the necessary needs of the patient.

The dialogue centered on trying to understand the patient’s reasons for refusing adequate care. They could assume that it was based on fear and anxiety, but they could also describe it in terms of the patient’s lack of knowledge or that the patient was unable to comprehend the medical situation they found themselves in. The professionals experienced that patients were unable to foresee the consequences of their own decisions. The reflections resulted in another ethical issue: to what extent is it acceptable to try to influence the patient to take part in the care offered, considered to be in their best interest; for instance, life-saving amputation or rehabilitation after hip fracture?

The discussions concerned how far it is morally justified to exert pressure on the patient. MCD participants were conscious of the patient’s right to influence care and also of their obligation to frame care according to patients’ wishes. They were concerned about the risk of being disrespectful in their eagerness to provide the best care.

For patients assessed as having adequate decision-making capacity, and who also gave good reasons for declining the help offered, the participants expressed less uncertainty. However, they expressed an obligation to further explore exactly on what grounds the patient had refused care. In one case, the staff discussed to what extent they should try to convince a patient that he must eat.

It feels like pieces of the puzzle are missing. Why has he made this decision? My intuition as a nurse tells me to promote health as much as possible. Of course he has self-determination, but why has he taken this decision? (Internal medicine).

### Struggling with standing up for the incapacitated patient’s nebulous interests

It was acknowledged that the family plays an important role in the process of framing care according to the patient’s wishes, but staff also discussed conflicts with families regarding the interpretation of these interests. Conflicts evolved around feeding, surgical procedures, and the need for more social services and support at home. Here, a role of advocacy and speaking up on behalf of the patient was described. Patients who were perceived not to be able to speak up for themselves because of advanced age, dementia or chronic illness, were sometimes seen as being unable to verbalize their needs and wishes. The professionals saw themselves as protectors of the patient’s rights, sometimes against family members’ perceptions, but also against the views of the home-help service. This role of advocacy seemed to be comprehended as an ethical demand on them, and as a part of their professional responsibility.

I think the thing with food is really difficult. I enjoy feeding people, but I don’t like forcing me on someone just because a daughter or a son wants it. (Geriatric rehabilitation).

There were reflections about uncertainties as to what it meant to do good for a seriously ill and older patient at the end of life who had limited possibilities to express their own wishes for care. Professionals struggled with conflicting purposes inherent in care; strictly palliative aims versus more life-sustaining aims, such as artificial nutrition or lifesaving amputation. One discussion concerned how the staff could do good for an old woman who rejected taking part in mobilization after a hip fracture.

She has lost the will to live. For how long must one fight with a patient who doesn’t want to live anymore and what’s to do good for this patient? (Geriatric rehabilitation).

### Coping with the conflict between using coercion to achieve good and protecting human dignity

In several of the patient cases that were discussed, the patient lacked decision-making capacity due to chronic or acute illness, and were unable to express their own wishes for care. Some patients in these situations also opposed care that was perceived by the staff as vital and necessary. In one case, a patient affected by dementia was running naked through the unit, refusing to be dressed:

It felt very difficult to leave her in that condition and we almost had to force her to put on dry clothes. It felt like that was a violation as well. It was very difficult to manage. (Internal medicine).

Participants were challenged on how to balance respect for the patient as a human being with doing good and preventing harm due to lack of care. In the case of the woman affected by dementia, staff expressed a concern for her health and wellbeing. Although many of these patients lacked decision-making capacity, professionals still experienced an ethical dilemma when they were challenged to fulfil important needs without the patient’s consent, when facing resistance, and, in critical situations, the use of coercion. Noticeably, the word “violation” was used to describe the experiences of the professionals when they felt morally forced to use coercion. One confused patient needed urgent urinary catheterization, and staff discussed how they felt obliged to physically hold the patient down to carry it out, something that ultimately relieved his pain. Nonetheless, this was experienced by staff as morally distressing and could be interpreted as an insult to his human dignity.

The term “violation” was also used to describe experiences of convincing patients with full decision-making capacity to undergo medical interventions, perceived by staff as necessary, such as an operation for vessel access preceding dialysis treatment. Patients’ rejection of care was seen as the hallmark of insufficient comprehension of their medical situation and needs, not as an expression of deliberately declining to take part in offered care. In these situations, participants expressed a responsibility to convince the patient about the necessity of the intervention, and, in some situations, they described applying quite harsh pressure, a strategy that participants sometimes experienced as a violation.

Then I felt that I have to push quite hard because it will not save her if she doesn’t get this surgery. She got her surgery later. Sometimes you almost have to do a little violation. (Dialysis care).

Although the staff defended their actions from a moral point of view, they were still concerned that the patients could experience their actions as disrespectful and as a threat to their human dignity, as well as to their autonomy.

## How to establish a responsible relationship with the vulnerable person

### Acknowledging the patient as a susceptible human being in a psychosocial context

Participants described the patient in the ethically difficult situation as vulnerable. They expressed awareness of the patient’s dependency on the staff and the patient’s psychological exposure by their loss of health and need for support. This situation required, according to the staff, a relationship built on trust and confidence.

The participants expected of themselves that they be in a position to read the patient’s state of mind. They wanted to pay attention to the various concerns and anxieties that go with being ill, and tried to identify patients who needed the support and presence of staff. In the following case, the staff discussed a patient who recently became dependent on dialysis:

I think she felt very exposed and she is used to be in full control of the situation. Now someone else had control. (Dialysis care).

Getting to know the patient was regarded as a valuable tool, as well as a prerequisite in the framing of care consistent with the patient’s needs and wishes. The participants aimed at viewing the patient’s health problem as a part of his or her entire life story, and regarded the patient’s psychosocial situation to be important in the delivery of good care.

Participants also acknowledged that patients had psychological and social difficulties that complicated their medical treatment. Diseases such as diabetes and kidney failure were seen as requiring significant effort from the patient, and the professionals experienced that factors such as a lack of social support from the family, economic difficulties and psychiatric illnesses, all influenced patients’ capability to take care of themselves.

### Protecting the patient’s dignity and integrity through practical measures in care

Participants considered it important to try to protect the patient’s dignity in the midst of illness, regardless of age or whether there was a need for technical procedures. Patients’ dignity was to be protected through the forming of daily care. Concern was expressed about patients being in what participants considered to be undignified situations, and they felt a responsibility to resolve or bring such undignified situations to an end. With the confused and naked patient, the staff felt obliged to dress her against her will:

To say that they have freedom of choice, do as you want, go around here in the hospital completely nude. Then I’d probably had felt more remorse for this situation instead, that I hadn’t done what I should have done. (Internal medicine).

The importance of protecting the patient’s integrity was discussed. Integrity was understood both as a physical and bodily concept and as a psychological concept of privacy. Bodily integrity could be challenged during procedures and interventions. Staff paid attention to patients who needed support in the protection of their physical integrity when undergoing reiterated treatments such as dialysis. However, conflicting purposes were experienced when they had to balance patients’ need for privacy with accommodations in single rooms and closed doors against their need of supervision for medical safety. Being a part of institutionalized health care was also perceived as a threat to the integrity and privacy of families, and the staff tried to balance protection of integrity and dignity with the patients’ needs of practical procedures and interventions.

Professionals saw the close and compassionate relationships between patients that took place as being important, but also viewed this phenomenon as a potential threat to patient privacy and integrity. Hence, some patients were regarded as being in need of protection against disturbances from other patients. Such protective measures were considered to be morally important.

### Defining personal moral responsibility towards the patient

Responsibility emerged as an important subject during the discussions. This concerned both professional and personal responsibility. The staff expressed a responsibility to fulfil the patients’ medical and nursing needs. This responsibility was a fundamental part of the professional role that many of the discussions proceeded from.

Well, I hope someone else could come by because I won’t find time myself. But she’s my responsibility today. (Geriatric rehabilitation).

How far this personal responsibility extended was a matter of debate, but so was the extent to which the general responsibility of the professional healthcare system reached. The staff expressed a will to help and support patients in difficult situations, but also felt a need to step back in situations where they failed to achieve better care for the patients. They described situations where they perceived it to be necessary to delimit their commitments. This was typically suitable when the patient had severe psychosocial problems and it was believed that the healthcare system had limited possibilities to help.

They also discussed the patient’s personal responsibilities. Typically, this issue emerged in the discussion when deliberating to what extent it was legitimate to try to influence the patient.

She has been fully informed. We can’t do anything more. She’s a grown-up human being. You could have that attitude as well and then let it go. (Dialysis care)

### Having patience to give the patient and family time to come to terms with illness and declining health

The dialogue evolved around patients who were rapidly declining in health. Older patients were deteriorating or facing death, and had increasing needs of support. Chronic illness, such as kidney failure, entailed deterioration with a need for continued treatment. It was acknowledged that patients and families needed time to become accustomed to new circumstances in life, the need for new treatments, or for moving to a nursing home. These needs seemed obvious to the professionals, however, the patient and family struggled with participating in the process due to what the staff saw as a lack of comprehension of the current situation. It was perceived that they needed more time to understand the new situation, and the staff regarded this as being morally important. This necessitated both patience and awareness of staff in order to wait for the patient and their family to come to terms with change.

I think we’re quite sensitive and try to adjust the hospital stay according to how far the family has come in their processing of the patient’s illness. And it is evident that if you have been married for 60 years and one is moving to a nursing home and I am staying at the house, it’s like a divorce. (Internal medicine)

## Discussion

The present study highlights the ethical conflict in everyday practice between comprehending the values and beliefs of patients and their families and framing the care according to professional responsibilities. The healthcare professionals’ moral reasoning can be likened to a see-saw of principle-based reasoning, undulating between their own professional convictions of what constitutes good care for the patient on one side, and the patient’s wishes and preferences on the other (Fig. 2). As the basis for the undulation, a relational-oriented reflection over a responsible relationship with the patient as a vulnerable person surrounded the dialogue (Fig. 2). The study shows that in MCDs that are conducted close to everyday care, principle-based and relational-oriented ethics complement each other. This is a significantly different composition than discussing hypothetical patient cases or patients not known to the participants, where relationships and emotions are often absent. Rasoal et al. showed, in the larger Euro-MCD project, how the MCDs departed from a sense of powerlessness, uncertainty and unease in the relationships with the patient and family (Rasoal et al. 2015), and how these feelings seem important for informing the moral reasoning process (Molewijk et al. 2011). The mixed-method article that complements the results discussed in the current article, shows that the need for the participants to talk about professional relationships and restoration of their own wellbeing, may also be essential in order to manage ethically difficult situations and thus eventually contribute to good patient care (Svantesson et al. 2017).

Unsurprisingly, the moral reasoning discussed in the present paper was infused with discussions about patient autonomy. However, these findings may help us to understand the professionals’ perceptions of patient autonomy in the midst of clinical practice, and may be interpreted through the framework of ‘relational autonomy’(Mackenzie and Stoljar 2000a). Autonomy, as it emerged from the liberal tradition, has been seen as a negative right to shape your life unhindered by others based on our own defined values (Jennings 2016). The moral agent, in this perspective, is both rational and independent, exercises free will, and has full moral responsibility for his or her actions (Materstvedt 2011). Furthermore, autonomy in the bioethical context has traditionally been equated with the ability to give informed consent to treatment or to decline such; a meaning of autonomy that originated from the field of research ethics (Dodds 2000). In the ideal health care service the patient and the professional meet each other on an equal level; the professional gives sufficient information, and the patient is free to accept or refuse treatment based on their own values (Donchin 2001).

In the present study, the professionals described their patients as being severely ill and in distress, all of which affected their decision-making capacity. Evidently, the ideal situation described above did not seem sufficient to help the professionals in facing the ethical difficulties, but the staff still had to deal with the patients’ decline, ambivalence or different kinds of unwillingness against the offered and, in their view, necessary health care. They strongly advocated that they could not leave the patient with their perceived insufficient and incorrect decisions which they thought would lead to considerable harm due to lack of care. They were at the same time morally troubled by the use of power to try to influence the patient and the risk of violating the patient’s dignity and integrity.

Contrary to autonomy interpreted in the traditional sense, ‘relational autonomy’ might serve as a better interpretative tool to understand the professionals’ struggles in the present findings. Feminist philosophers have been criticizing the traditional concept of autonomy and what is taken to be its failure in contributing to the interpretation of autonomy in health care (Donchin 2001; Jennings 2016; Pullman and Hodgkinson 2016). Feminist philosophy has claimed that the traditional view on autonomy is based on a masculine character, ideally capable of living a self-sufficient, isolated and independent life separated from others (Mackenzie and Stoljar 2000a). This conception stands in opposition to the primarily feminine experiences of the care of, as well as the dependency on, others, and has been perceived as a devaluation of women’s experiences and the values arising from them (Mackenzie and Stoljar 2000b).

*Relational autonomy* has been suggested as an alternative framework for the understanding of autonomy in clinical practice (Dodds 2000). The concept departs from the relational-oriented ethics of care, and captures the need to frame the patient’s autonomy within a responsible relationship with others (Mackenzie and Stoljar 2000a). According to ethics of care, human beings are vulnerable and dependent upon one another; we are deeply situated in relation to other people and, consequently, this is the starting point for an alternative approach to autonomy as well as to our moral life as a whole (Pettersen 2011). Instead of framing autonomy in terms of a negative freedom, relational autonomy is described as the positive freedom to make judgments according to one’s own values and goals (Verkerk 2001). Relational autonomy demands engagement from professionals to support and promote a patient’s autonomy in making judgements that are true to their own wishes and values (Mackenzie 2008; Widdershoven and Abma 2011). Threats to patient autonomy mainly come from abandonment by the professionals, not because of interference, in the decision-making process.

This means that the professionals in the present findings would not leave their patients to make decisions which they judge as not being in the patients’ best interests. But even more important, they struggled with respecting the decisions of the patients, which they were not convinced were made truly according to the patients’ wishes for their health and future. The professionals experienced that the patients refused necessary care due to anxiety, distress or confusion, and not due to careful considerations based on adequate decision-making ability. They felt that their responsibility was to not leave the patient to deal with the consequences of their inadequate decisions, but instead, through learning to know their patients as persons and by striving to capture the patients’ perspective, to try to guide the patient to good care. They struggled to understand the processes the patient and their families went through when they suddenly became ill or when the chronic illness worsened, and with how to work through the individualizing of support.

A key aspect of relational autonomy, and as shown in the present study, was to acknowledge and establish a responsible relationship with the vulnerable person. Vulnerability has been seen as both something that encompasses all humans as we through life experience illness and injury, as well as loss and grief, but the view that some humans are more vulnerable than others, and hence need extra attention and protection, has also dominated (Martin et al. 2014). The professionals in the present study considered the patients to be especially vulnerable due to their loss of health and need for support, but also due to their physical circumstances, such as undergoing hospital care and coming to terms with new circumstances in life. Traditionally, vulnerability and autonomy have been seen as contradictory phenomena (Mackenzie et al. 2014). Vulnerability is linked with helplessness, neediness and victimhood; hence, the protection of vulnerable people has been viewed as an open door to paternalistic and coercive forms of intervention (Christman 2014). The professionals struggled with the interpretation of the patient’s wishes and preferences for care, but while concurrently striving to accomplish what they interpreted as satisfactorily medical and nursing care for a vulnerable human being.

The balance appeared to tilt in favour of the professionals’ convictions of what *they* conceived as the patient’s best interest and they could be regarded as displaying a paternalistic attitude in claiming to know what is best for the patient (see Fig. 2). Paternalism is understood as interference, either against one’s will or without one’s consent, in the aim of promoting the good, and is in general not ethically justifiable (Materstvedt 2011). Weaker forms of paternalism might be justifiable in situations where the patient’s ability to display autonomy is significantly impaired due to acute or chronic illness, as observed in the present findings (Christman 2014). In spite of this, and perhaps surprisingly, the professionals experienced moral distress when trying to convince patients to partake in measures and interventions which they judged to be in the patients’ best interests. Noticeably, they frequently employed the strong notion of “violation” to describe their moral feelings in the interaction with patients. Although the professionals considered their actions to be ethically justifiable, they nonetheless described the moral distress of the patient’s situation in the encounter. The patients were not present during the MCDs and this might be seen as a weakness of the MCD method. However, in the present study, the facilitators influenced the reasoning by trying to adopt the perspective of the absent patient and their family. In interviews with these facilitators in a previous study, they themselves pointed out the importance of being the patient’s voice, as well as a need to challenge the healthcare professionals’ homogenous reflections. Their challenge of simultaneously adopting the disparate roles of being accommodative and challenging seemed to enhance their moral reasoning as well as restoring the participants’ sense of wellbeing (Rasoal et al. 2017).

## Conclusion

Moral reasoning in MDCs is apparently infused by discussions about providing autonomy for vulnerable patients. In theory, promoting respect for a patient’s autonomy is quite uncontroversial, but, to deal with autonomy in everyday care is far more complicated. The vulnerable severely ill patients’ diminished capacity, together with healthcare professionals’ responsibility to secure good care, demonstrates a need for a relational approach to autonomy. Autonomy for the vulnerable patient can only be achieved by interaction and engagement, rather than abandoning the patient to decide for themselves. Although the conception of relational autonomy cannot solve all ethical problems, relational autonomy is helpful for understanding healthcare professionals’ extended responsibility to learn to know their patients through their life stories, and to enable them to frame the care according to the patients’ most profound wishes and values, that is to say, to practice person-centered care.
